# Photobiomodulation and Oral Mucositis: A Systematic Review

**DOI:** 10.3390/dj8030087

**Published:** 2020-08-05

**Authors:** Mark Cronshaw, Steven Parker, Eugenia Anagnostaki, Valina Mylona, Edward Lynch, Martin Grootveld

**Affiliations:** 1 Leicester School of Pharmacy, De Montfort University, Leicester LE1 9BH, UK; thewholetooth@easynet.co.uk (S.P.); eanagnostaki@densindente.de (E.A.); val.mylona@yahoo.com (V.M.); edward.lynch@hotmail.com (E.L.); mgrootveld@dmu.ac.uk (M.G.); 2 School of Dentistry, College of Medical and Dental Sciences, University of Birmingham, Birmingham B5 7EG, UK; 3 School of Dental Medicine, University of Nevada, Las Vegas, NV 89106, USA

**Keywords:** cancer, chemotherapy, LED, laser, LLLT, low-level laser therapy, oral mucositis, photobiomodulation, stomatitis, radiotherapy

## Abstract

Oral mucositis (OM) is a debilitating complication of chemotherapy, and head and neck radiotherapy. In an effort to offer the best possible advice within the limitations of published research, a systematic review with an extended discussion and commentary on dosimetry and dose delivery is presented. Using keywords as listed, Pubmed, Google Scholar and Cochrane databases were searched during a period extending from 1995 to 2019. A total of 782 abstracts were identified. A total of 50 papers were analysed, and of these, 29 satisfied criteria required for systematic review in accordance with an optimized PRISMA statement. Clinical outcome as reported was subject to analysis with respect to time of intervention, incidence and severity of oral mucositis, and pain amelioration, and a comprehensive combined univariate and multivariate statistical analysis of the methods employed was performed. Recommendations are made with respect to the timing of the intervention. Moreover, there is an extended discussion available on the treatment care rationale of photobiomodulation (PBM), and its adjunctive association with OM. In conclusion, early prophylactic application offers clear advantages in clinical management. The many studies and associated variables and covariables assessed here revealed a choice of delivery techniques, associated wavelengths and many further indices to consider with regard to the accomplishment of optical parameters. It is therefore our recommendation that clinicians use PBM as a therapy with a full and proper understanding and training in order to optimise the clinical effects achievable.

## 1. Introduction

Oral mucositis (OM) is a common consequence of chemotherapeutic drug infusion, as well as head and neck radiotherapy (HNRT). A few days after such treatment has commenced, the patient complains of soreness, and the oral tissues appear red and smooth. Rapidly, the integrity of the mucosa breaks down and ulceration occurs to affect the buccal mucosa, ventral lingual mucosa, soft palate and the inner aspects of the lips and the floor of the mouth [1]. Unfortunately, associated pain is intense, and OM can severely impair oral functions including speech and feeding. This may lead to a requirement to discontinue cancer therapy, despite the increased risk of treatment failure. Treatment strategies may include concepts of pre-chemo-radiotherapy and the prescription of targeted local and systemic measures; however, such strategies can be viewed as equivocal and often unpredictable. Hence, the development of sub-ablative photobiomodulation (PBM) as a possible therapy has been a succession of initial elemental application, with enhanced sophistication leading to a greater emphasis on wavelength choice and dose specification.

The serious morbidity that can arise from this clinical problem is associated with extended hospital-based care needs, together with a high degree of patient pain. Last year, following extended multi-centre clinical trials and multiple systematic reviews, the joint task force of the Multinational Association for Supportive Care in Cancer (MASCC), and the International Society of Oral Cancer and Oncology (ISOO), agreed new updated guidelines [2].

OM occurs in over 80% of patients undergoing haematopoietic stem cell therapy (HSCT), in addition to a high incidence in head and neck radiotherapy (HNRT). OM is also associated as a common complication (40%) of the management of many other types of cancer chemotherapy treatment [3,4]. The pathology of OM is associated with pro-inflammatory pathways consequent upon the chemotherapeutically and radiotherapy-induced disruption of basal cells of the oral surface epithelium. Subsequent infection of the exposed dermal layers elicits an acute inflammatory response via increased cyclo-oxygenase production of prostaglandin E2, an upregulation of nuclear factor kappa B and interleukin 6, histamine release, and increases in the production of bradykinin and substance P [5]. This results in severe pain and swelling, which can be difficult to manage in already debilitated patients.

## 2. Photobiomodulation Therapy

Photobiomodulation therapy (PBM), formerly known as low-level laser therapy, is the application of lasers or non-coherent light sources such as LEDs to beneficially influence cellular metabolism. It represents a non-thermal treatment, and the energy and power levels associated with this therapeutic regimen are below the threshold associated with adverse heating effects or mechanical cellular damage.

Following many years of research conducted in vitro, animal studies, and more recently human clinical trials, have offered a considerable amount of information regarding the mechanisms associated with PBM, and the benefits that may be gained in clinical practice [6,7]. The principle of operation is the transfer of incident photonic energy to a cellular target, which then affects intra-cellular organelle metabolism. It is considered that one of the primary targets for this form of therapy is the mitochondrion, which respond to the absorption of red to near-infra-red (IR) wavelengths of light by an increase in activity of the electron transport respiratory chain. This results in an increase in the production of ATP, as well as nitric oxide (NO^●^), and there are complex downstream effects on gene expression, which give rise to many changes beneficial to cellular metabolism [8]. For example, in view of the change in the redox status of the cell into an aerobic cycle of activity, cells are less susceptible to stress-induced apoptosis [9]. Furthermore, there is the selective uptake of pro-inflammatory cytokines and an inhibition of COX-2 activity [10,11,12]. NO^●^ is a vasodilator associated with increases in the perfusion of tissues with oxygenated blood; the lymphatics also become dilated and less porous, which results in a satisfactory resolution of swelling [5,6]. Furthermore, there is the increased production of pro-collagen and growth factors including, for example, vascular endothelial growth and fibroblast growth factors [12,13,14,15,16]. There is an increase in cellular motility and rate of division, which further promotes wound resolution. An additional benefit may be the marked reduction in nociception consequent upon the elimination of the acute inflammatory mediators associated with heightened axonal activity, as well as some selective inhibitory effects on axonal transmission [17,18]. Finally, researchers consider that PBM can influence cytoplasmic reactive oxygen species (ROS) levels which are normally associated with the glycolytic anaerobic cycles of metabolism [19], and there appears to be an enhanced immune response in the local region of the OM. However, the science associated with the mechanisms of PBM is still under investigation. Although much is known regarding the extraordinarily complex cellular pathways of activity, PBM appears to exert far-reaching effects on many cellular sub-systems and organelles, including the endoplasmic reticulum, cellular membranes and the nucleus [5,20,21].

The aim of this systematic review is to analyse the very many variables of parameters applied and the timing of the intervention in order to offer clinicians an indication of the most likely optimal techniques and parameters required to achieve clinical success.

## 3. Materials and Methods

Databases Pubmed, Google Scholar and Cochrane were searched for the keywords “Photobiomodulation”, “PBM”, “Low level”, “LLLT”, “LED”; “Cancer”; “Stomatitis”; “Chemotherapy”; “Radiotherapy” AND “Oral mucositis” for a period extending from 1995 to 2019. A total of 782 abstracts were identified from January 1995 to December 2019.

Our inclusion criteria were human clinical controlled randomised studies, and retrospective case analyses. Detailed data extraction from the expanded scope of papers included were subjected to data analysis as described below in order to identify significant trends, and 50 studies were read in full text and screened in accordance with the PRISMA protocols. A total of 29 studies were included for the purpose of an updated systematic review [22]. Exclusion criteria for this narrower sub-range of studies included the absence of blinding, no randomisation nor the use of a control group. Moreover, non-English publications and reports not subjected to accepted evidence-based peer reviewed journals were also excluded. Aside from the databases described above, the authors also conducted a manual search of key references featured in the included papers to identify any missed by the primary search methodology.

**Figure 1:**
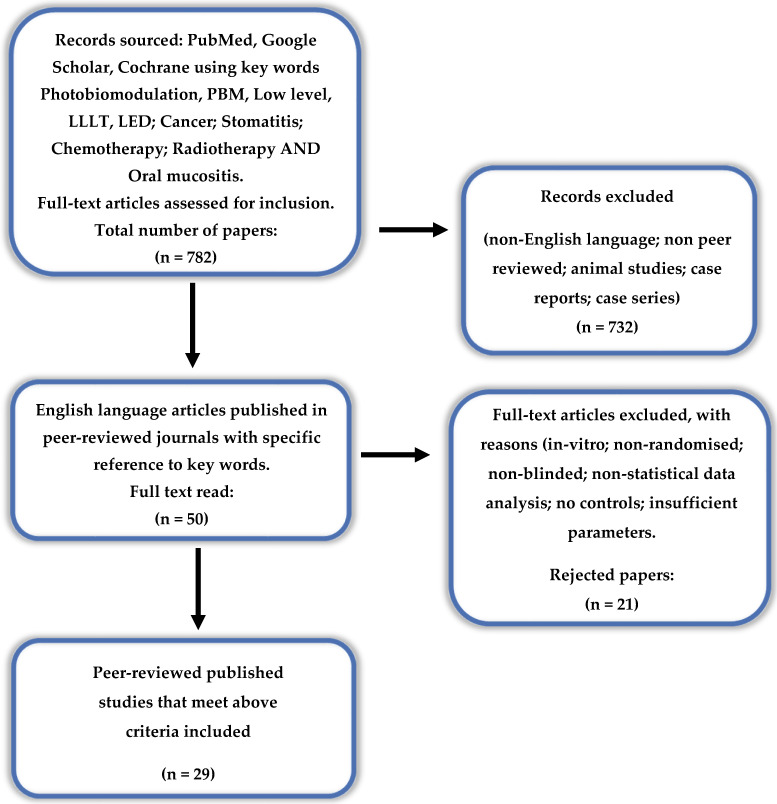
PRISMA flow-chart of selected criteria for the included article reports. (from [22]).

Outcome scores for reductions in the incidence and severity of OM, and a separate score for pain reduction were made. A null effect was scored as zero, and a statistically significant score in the range up to 20% of the corresponding control group was scored as 1. A 20–40% improvement was scored as 2, and a ≥ 40% improvement observed was scored as 3 (Figure 1).

The eligibility criteria according to the PICOS process have been interpreted as follows:

- Population = patients with oral mucositis;
- Intervention = chemotherapeutic and palliative measures + PBM laser therapy;
- Compared with = chemotherapeutic and palliative alone;
- Outcome of interest = pain; function QoL;
- Study type = randomised controlled trials.

Furthermore, for the selection of eligible articles, a grade scale for quality assessment was applied based on the following criteria:

- Randomisation and blinding;
- Comparability of groups at baseline (e.g., severity of disease);
- Description of treatment and irradiation protocol;
- Clinical assessment at baseline and at follow up—VAS, visual assessment improvement in function.

The selected papers were subject to a risk of bias using a modified Cochrane ROB analysis. Analysis was by three of the authors randomly considering the articles and arriving at consensus as to level of scientific rigor of the published data. Following this assay, all the selected papers were considered medium to low risk in terms, scoring between 7 and 10 against the series of selected risk criteria.

The experimental design for the univariate analysis of the abilities of all possible predictor variables, both qualitative and quantitative, to exert a significant effect on laser treatment outcome (scored 0–3 as detailed above), primarily involved a univariate analysis-of-covariance (ANCOVA) model, which incorporated 2 qualitative and 13 quantitative predictor variables. No interaction effects were explored in view of limitations with sample sizes (n = 27 only) and hence degrees-of-freedom available for hypothesis testing. The qualitative sources of variation were pre- and post-treatment application options, and the class of treatment, whereas the quantitative ones comprised wavelengths, powers, applications times, numbers of both sites and points, number of days involved and frequency of application, joules (J) delivered and total J delivered, J/cm^2^, W/cm^2^, beam diameter (mm) and area (cm^2^), all of which were fixed effects. ANCOVA was conducted with the *XLSTAT2016* software with each of the above possible predictor variables analysed individually. Data variables were weighted with the total number of participants included in each study explored. For statistical significance, a Bonferroni-corrected *p* value was set as a significance level, which in this case was 0.05/15 = 0.0033. 

Subsequently, multivariate (MV) statistical analysis was performed with the partial least-squares regression (PLS-R) technique, where the dependent variable was treatment outcome, and the potential explanatory ones were the 2 qualitative and 13 quantitative ones specified above. Quantitative variables were mean-centred and auto-scaled prior to analysis, including the dependent clinical outcome score one. This MV strategy was also performed using the *XLSTAT2016* software. 

For both types of analysis, missing values were estimated for selected predictor variables by replacing them with their mean column variable values, and then automatically reducing the statistical model’s power (and correspondingly degrees-of-freedom in the case of univariate ANCOVA analysis) accordingly [23,24]. 

## 4. Results

The majority of studies identified and included in this review applied visible red wavelengths within the 632.5–660 nm range. In addition, other studies applied wavelengths within the range of 780–970 nm. Most of the studies included employed a laser system, whereas a small number utilised an LED with some positive results reported. The majority of the studies used a small optical spot size of only 0.04 cm^2^ with a probe in contact with or near-contact to the target tissues. The number of points of application for these studies varied from 15 to 80, with a declared fluence at each point of 1 to 83 J/cm^2^, and an irradiance of 0.024 to 13.8 W/cm^2^.

**Figure 2:**
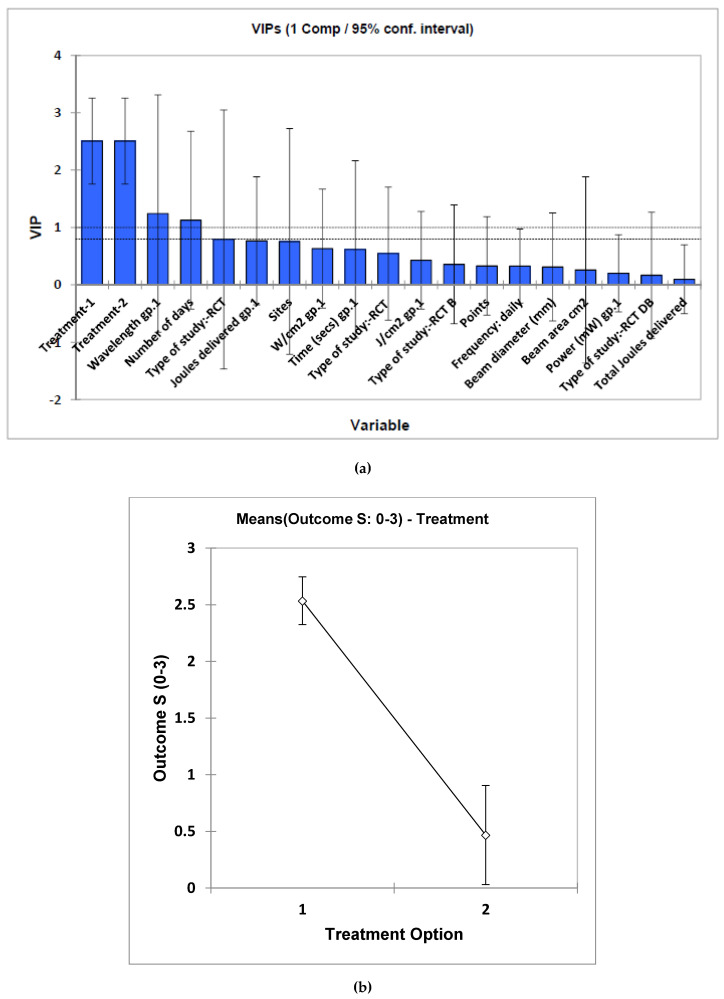
(**a**), Estimated mean ± 95% confidence intervals (CIs) for component 1 variable importance parameter (VIP) values for each potential “predictor” variable included in the multivariate PLS-R analysis model applied to the sample size-weighted systematic review dataset. VIP values are considered statistically significant if the lower 95% CI is >1.00, and therefore only treatment option (1 or 2) was found to be a significant contributory variable towards clinical outcome score. (**b**) Plot of mean ± 95% CI values for the two treatment options obtained from the univariate ANCOVA of the full dataset. Treatments 1 and 2 correspond to pre- and post-laser treatment options, respectively.

Outcome results included reductions in VAS, along with the incidence and grade of severity of the OM. The most positive outcomes were reported in those studies which offered pre-treatment followed by concurrent support to the cancer treatment modalities of chemotherapy and/or radiotherapy. MV analysis of these data by PLS regression, which were participant size-weighted, revealed that this pre-/post-treatment approach was the only one which significantly determined treatment outcome (variable importance parameter 2.51 ± 0.75 (mean ± 95% confidence intervals)), the pre-treatment regimen providing a high level of improvement (Figure 2a). Similarly, univariate analysis using ANCOVA also performed on the participant size-weighted dataset confirmed a high level of distinction between these pre- versus post-treatment criteria, again with the pre-treatment selection providing a much more satisfactory outcome (*p* = 3.19 × 10^−7^). However, the beam area variable was also univariately statistically significant, with *p* = 7.35 × 10^−4^. Mean ± standard error (SEM) clinical outcome parameters for the pre- and post-treatment applications were 2.54 ± 0.15 and only 0.47 ± 0.40, respectively, values also determined on the study sample size-weighted dataset (Figure 2b). All further potential explanatory variables were found not to be statistically significant when evaluated in this univariate analysis model.

In studies which combine the analysis of datasets via both univariate and MV techniques, it is quite well known that predictor/explanatory variables which are significant in a univariate testing model may also remain insignificant in an MV context [1]. Notably, in the MV PLS-R model applied here, the beam area variable was found to be insignificant, but it was so when tested univariately in our ANCOVA model. Such an observation may be accounted for by the “masking” of such predictive information from this variable by the quite large number of remaining, albeit uninformative (poorly predictive) variables, i.e., 13 in this case. Such statistical complications, which are encountered quite frequently, are linked to the availability of reliable estimates of covariances, particularly when there are large numbers of potential predictor variables and relatively small sample sizes, and/or a poor correspondence between the univariate and MV testing strategies involved here.

A detailed description of the complete set of data extractions and parameters applied, plus a digest of associated information are compiled in Table 1. As noted above, 29 papers fulfilled the defined limits of this systematic review. 

## 5. Discussion

The defined optimal outcome found by our analysis of a result by pre-treatment and by the application of a larger spot size has important implications for clinicians. Reduced incidence and severity of OM reduces patient morbidity and considerably simplifies patient management. OM is a painful and distressing condition for patients and any reduction in this greatly enhances the patient’s comfort, and reduces the need for concomitant analgesics and wound management. The finding that a larger surface beam area achieves an optimal result is a clear indication that this method be preferred as opposed to in contact techniques using a small optical guide. 

With regard to the benefits offered by PBM in the management of OM, the issue may be regarded as a number of separate problems: firstly, that of the prevention of the cellular disruption which results in the major clinical problems caused by painful oropharyngeal ulcers associated with the inflammatory response; secondly, that in the presence of varying degrees of severity of ulcers or radiodermatitis associated with combined chemotherapy/radiotherapy, healing is impeded consequent to the systemic debilitation associated with the intervention, secondary infection of the exposed wounds and/or radiation-induced local tissue damage; thirdly, that the consequence of the resultant peri-oral and pharyngeal ulceration may generate significant pain and inflammation. Aside from the immediate marked discomfort, this can also interfere with feeding and speech activities and congruencies, which further complicates and extends patient care requirements.

The most recent systematic review by the Mucositis Study Group of the Multinational Association of Supportive Care in Cancer/International Society for Oral Oncology (MASCC/ISOO) revised and extended the range of clinical applications of PBM in the prevention of OM. Based on the expanded literature, it is apparent that PBM can offer benefit to many patients. 

Following the 2018 WALT (Nice, France) meeting, there was general agreement in accordance with the current consensus opinion across many clinical applications, including the treatment of OM, that the optimum dose for many treatment modalities was ca. 5 J/cm^2^ at the cellular level, with a recommended low rate of energy delivery (irradiance) of between 10 and 150 mW/cm^2^ [6,7,8,52,53]. Indeed, a suggested upper limit of 6 J/cm^2^ at the target tissue level in the management of OM had previously been proposed based on the requirement to avoid phototoxicity consequent to hyperthermia [54,55]. In accordance with the views of Elad et al., our analysis supports the view expressed that these parameter ranges were based upon clinical efficacies reported in the literature, rather than upon clinical safety data [56]. 

In this analysis, the choice of wavelength range applied to achieve a positive effect has been found to be 632.8–970 nm. This choice of wavelength appears to be a reflection of those chosen for the studies conducted to date, and it is the opinion of the authors that, based on the more general published research on PBM, wavelengths within the range of 600–1064 nm may be effective. As for choice of source, there is supportive evidence that for some superficial conditions, a non-coherent light source such as an LED may be effective as an alternative to a laser delivery system [57].

Indeed, it is noteworthy that 14 out of the 29 studies included here employed a small optical spot size of 0.028 to 0.04 cm^2^. Furthermore, the range of the number of points of application used in these studies was from 15 to 80, with a treatment time in sum of 320–1350 s. The radiant exposure (fluence) was in the range of 1.4 to 107 J/cm^2^, with an irradiance of 0.0042 to 13.88 W/cm^2^. The total energy delivered amounted to a range of 10.75 to 216 J. An alternative approach is to use a larger optical “footprint”, and this may offer a number of benefits. Point source devices limit the area and volume of tissues treated and add to the complexity of the therapy as a transferrable skill. While it is straightforward to identify and treat lesions, covering large areas for prophylaxis is easiest practically using a large optic beam. Given the advent of collimated non-contact hand pieces for PBM, these would appear to offer distinct operator advantages over the contact placement of fine diameter optic probes. In this regard, it is noteworthy that the finding of statistical evidence for the treatment of a larger area is very interesting. Our contention is that the use of a large optical beam spot size and the application of the same to an extended area covering the entire visible oro-pharynx are advisable.

However, given these highly variable parameters, it is entirely understandable that non-laser specialist clinicians may fail to understand the meaning of this with respect to treatment delivery options.

**Figure 3:**
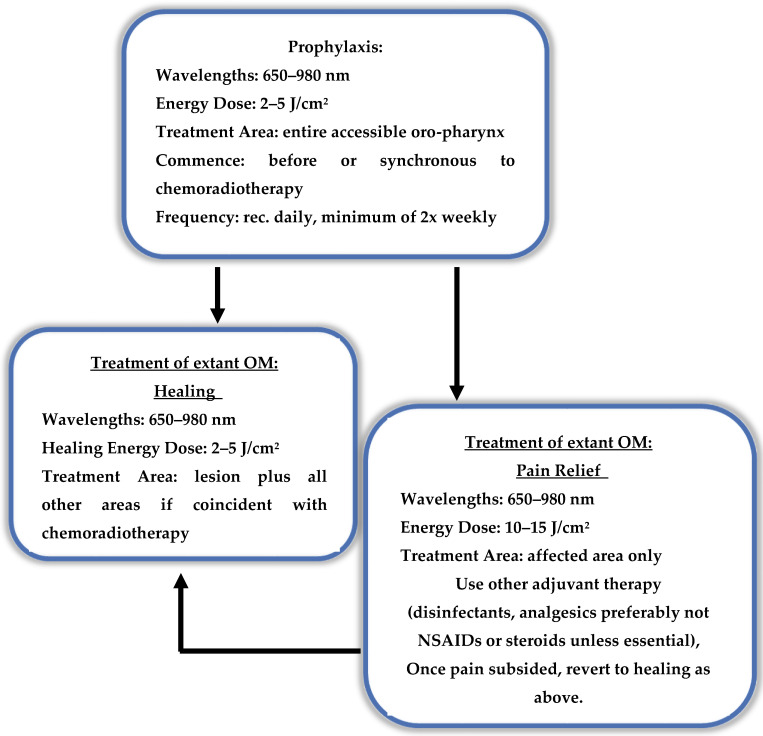
Proposed treatment decision tree—pain or healing? Outline of applied photobiomodulation (PBM) dose parameters to address the needs of pain relief and healing.

It is therefore essential to have a broad appreciation of laser–tissue interaction to be able to make relevant deductions from previously published research. In general terms, to successfully model an optimised protocol, a decision tree will be required, specifically one which determines the output power, time of application and frequency of therapy (a proposed decision tree is shown in Figure 3). 

Consistent with the expert consensus paper by Zadik et al. [2], we find that the most effective and consistent strategy in the management of OM is prevention rather than mitigation and correction. It is our view that this is an important point worthy of emphasis, specifically that the optimal time to intervene is prior to the administration of CT and/or RT, with a follow-up coincident with that of the active cancer therapies. 

The associated processes in the prophylaxis of OM are also worthy of discussion. The pre-conditioning of tissues and organs prior to medical intervention has a long pedigree, and the processes linked with these phenomena have been the subject of considerable literature debates [9]. By virtue of pre-stressing tissues, it is possible to increase their capacity to survive the associated insult of surgery, in addition to their susceptibilities to oxidative stress episodes. Cells have also been found to be more resilient and better able to withstand chemical and radiation insult from pre- or post-exposure to light. Indeed, it is recognised that PBM can have dose-related effects, such that at a range of 2–8 J/cm^2^, there is an optimal potentiation of the electron transport chain (ETC) to operate in a highly productive cycle of aerobic metabolism. One major consequence of PBM is the optimal biosynthesis of ATP and NO^●^, which occurs in cells which are physiologically more resilient and more able to tolerate stress induced either by exposure to cellular anti-metabolites (chemotherapy), or to hard radiation. Furthermore, at higher levels of photonic exposure, light-induced cellular protective systems can be activated, which can again confer some survival benefits to cells subsequently exposed to stressor systems [6,58,59].

Elevated production of ROS is associated with both low and high levels of activation of the ETC. At medium-to-higher levels of production, ROS are associated with the activation of NFkB and Nrf2KEAP and also stimulate HIF-1alpha’s ligand [19,21]. However, downstream effects include the triggering of mitosis, together with the production of important growth factors such as VEGF and TGF-B. This is, however, also associated with the activation of nuclear transcription factors associated with a pro-inflammatory pathway. In addition, high levels of ROS activate the uncoupled protein response, which results in decoupling of the ETC, a reduction in the inner mitochondrial membrane potential and back-flow from the inner mitochondrial space of protons into the mitochondrial matrix [60,61,62]. This results in the loss of the electromotive and proton flow force required to drive the membrane-bound turbine at unit 5 of the ETC, and there is therefore a progressive reduction in mitochondrial ATP manufacture.

In an elegant in vitro study of phototoxicity, the effects of high levels of incoming photonic energy and elevated temperature were investigated by Khan et al. [58]. High cytoplasmic levels of ROS were identified as an activator of a hormetic stress response via ATF-4 and HSP70, which can serve to protect cells against damage. ROS levels can, however, rise beyond the immediate shielding capacity of cellular systems, and this gives rise to elevated levels building in the cytosol and the mitochondrial matrix. Should the levels of ROS continue to increase, these may prove to be highly toxic.

The natural regulators of mitochondrial activity are central to many disease processes, and there is a considerable body of research that has investigated this in relation to both health and disease. Many aspects of cellular physiology are amenable to manipulation by virtue of photonic interactions to the cellular apparatus. Apart from the above described processes associated with the ETC, PBM also affects important membrane-bound ion channels known as TRPV’s [24]. In addition, it is highly probable that there are many other photoreactive targets for PBM, which in total can exert profound effects on cellular activity. There is, however, a dose-related outcome, since at lower levels of photonic exposure, photobiostimulation may be induced, whereas at higher levels there can be photo-induced cellular inhibition. In the scientific literature, this is referred to as a biphasic response; however, since photo-activated inhibition is associated with analgesia, it has been proposed that it may be more clinically useful to regard this as a multiphasic response process [62,63]. 

In respect to OM, the dosimetry recommended for prophylaxis fits into the range associated with photobiostimulation, i.e., 2–8 J/cm^2^. At higher levels of dosimetry, in accordance with the principle of pre-conditioning stress, it is in principle possible that as a prequel to CT or HNRT there may indeed be some clinical gain. However, to date, this possibility has not been adequately explored.

With healing, PBM is clearly an effective measure in wound resolution associated with OM. The mechanisms associated with this process include the selective inhibition of pro-inflammatory cytokines, an improved aerobic blood supply to the base of the wound, enhanced drainage of inflammatory exudate via the lymphatic chain and the promotion and stimulation of the immune system [5]. Again, the dosimetry associated with the promotion of wound healing using PBM is well known. An added complication of OM is infection of the exposed wound and in this regard, investigations exploring the potential for antibacterial photodynamic therapies and the adjunctive use of anti-bacterial agents are clearly important aspects of an overall controlled wound management regimen.

**Figure 4:**
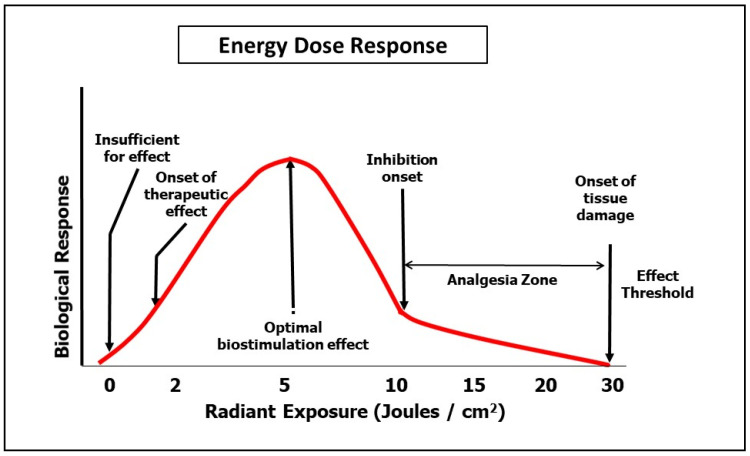
Multiphasic dose response.

There is at present no consensus on PBM protocols for pain management in OM. Our analysis here of published research on pain amelioration following PBM with OM revealed a general agreement in the literature that this was an evidence-based outcome. However, the size of the effect was relatively small, and offered at best a moderate clinical gain. Beyond the management of OM, there is an extended range of applications in pain management which have attained acceptance to the levels of multiple systematic reviews, for example, in relation to some musculo-skeletal disorders [64,65]. Following a systematic review by Kate et al. on PBM parameters associated with pain management, a number of bands of dose were identified, all of which were beyond the range of therapeutic doses normally applied in OM management [66]. This was clearly appreciated by Simoes et al. who applied 10 J/cm^2^ at a delivery power of 1.0 W as a pain relief PBM dose, which was recorded as beneficial, although the healing associated with the OM was subsequently found to be slower than that found at a lower applied dose [67]. With regard to practical applied clinical management protocols, the judicious application of higher dose PBM to achieve analgesia may offer some temporary amelioration of the severe pain associated with the condition. In combination with other means of managing the wound such as topical and systemic medication, PBM-induced analgesia may offer some palliative gain as a short-term measure. In our opinion, the decision to adopt the same strategy ought to be balanced by the knowledge that wound healing is best served with low dose PBM as a follow-on beyond the immediate acute episode. Based on the findings of Kate et al., a suggestion is made here of a dose of 10–20 J/cm^2^ to the area of the lesion. Further studies must, however, be conducted before this proposal can be accepted as a validated evidenced-based clinical protocol for this condition. Cellular systems are very sensitive to over- or under-dosage, since too little results in a null effect, as does too much, albeit with higher energy delivery, within the 10–30 J/cm^2^ range, which nevertheless appears to represent an optimal level for analgesia. This is, however, an area with an emerging evidence base, and in the authors’ opinion, it is of paramount importance not to subject tissues to a thermocline in excess of 43 °C for fear of ascending phototoxicity [58] (Figure 4).

Lasers have an inherent potential of deep tissue penetration in view of the physical property of coherence since they are a more intense light source [67,68,69,70,71]. Further, there can be a beneficial increase in energy into deeper tissues using lasers consequent to deep tissue photon collision and amplification (“speckling”) [72,73]. A further point of note relates to the optical beam shape, since there is the recognition that the inherent Gaussian beam profile associated with fibre-delivered laser systems can result in an energy distribution which peaks within the mid-third of the beam [74]. 

Newer “flat top” profile (uniform cross-sectional fluence) laser hand-piece attachments are becoming available, and these appear to offer some significant potential operator benefits: aside from rectifying the beam to permit a more uniform energy delivery; indeed, a flat top profile of surface energy delivery is more efficient in penetrating more deeply into tissues in the red to near infra-red wavelength regions using a laser system [75].

## 6. Conclusions

Taking into consideration the extended supportive evidence base, PBM for OM is a safe and effective measure to mitigate OM associated with chemotherapy and HNRT. However, the science and evidence bases have not as yet provided a consistent set of multiple study meta-analysis-based determinations to provide a wide range of optimised clinical protocols. Pre-conditioning and concurrent prophylactic therapy are optimal in results. The coverage by a larger beam area of the tissues is supported. It is recommended that in pain management, a choice is made between the amelioration of pain and the optimisation of healing, and suggested protocols are presented here for continued evaluations based on the best current supported research. There is the evident need for continued research, and as an aid to the same, it is hoped that this study with its extensive data extraction may prove to be of assistance.

## Figures and Tables

**Table 1:** Published articles by name and reference with associated data.

Author [ref]	Type of Study Pre- or Post-Treatment	Test T/Control C Group Treatment Tx Delivered (χ = Unknown Value)	Light Therapy Parameters (χ = Unknown Value)	Outcome VAS/S (sig.) (χ = Un-Known Value)	Comments RT = Radiotherapy CT = Chemotherapy
Barasch, 1995 [25]	RCT DB Post-treatment	20 Patients. Split mouth. Tx delivered 1 x day x 5 days. 5 sites @ χ points.	λ632.8, CW, near-contact, 25 mW, fluence 1.0 J/cm^2^, Irrad. 0.04 W/cm^2^, total energy 28 J, Beam dia 10 mm, 0.8 cm^2^, 40 s	1/2	RT + CT Area treated 28 cm^2^
Cowen, 1997 [26]	RCT DB Pre-treatment	30 Patients. T—15, C—15. Tx delivered 1 x day x 5 days. 5 sites @15 points.	λ632.8, CW, Spot 1 cm, 60 mW, fluence 1.5 J/cm^2^, Irrad 0.06 W/cm^2^, total energy 45 J, Beam dia 1.2 mm, 1.0 cm^2^, 10 s/point	1/3	RT + CT Area treated 75 cm^2^
Bensadoun, 1999 [27]	RCT DB Pre-treatment	30 Patients. T—15, C—15. Tx delivered 1 x day x 35 days. χ sites @9 points.	λ632.8, CW, Spot 1 cm, 60 mW, fluence 2 J/cm^2^, Irrad 0.06 W/cm^2^, total energy 17.8 J, Beam dia 1.2 mm, 1.0 cm^2^ 33 s/point	1/3	RT Area treated 9 cm^2^
Antunes, 2007 [28]	RCT B Pre-treatment	38 Patients. T—19, C—19. Tx delivered 1 x day x χ days. 9 sites @135 points.	λ660, CW, Spot 1 cm, 46.7 mW, fluence 8.9 J/cm^2^, Irrad 0.0042 W/cm^2^, total energy 33.75 J, Beam dia 1.6mm, 0.028 cm^2^, 16.7 s/point	1/3	CT
Arun, 2006 [29]	RCT DB Pre-treatment	50 Patients. T—25, C—25. Tx delivered 1 x day x 5 days. 3 sites @ χ points.	λ632.8, CW, 10 mW, fluence 1.8 J/cm^2^, Irrad. χ W/cm^2^, total energy 5.4 J, Beam dia χ, χ cm^2^, 180 s	2/3	RT
Schubert, 2007 [30]	RCT DB Pre-treatment	70 Patients. T(i)—23, T(ii) 23 C—24. Tx delivered 1 x day x 10 days. 6 sites @ χ points.	T(i) λ660, CW, Spot 1 cm, 40 mW, fluence 71 J/cm^2^, Irrad. 0.04 W/cm^2^, total energy χ J, Beam dia 1.6 mm, 0.028 cm^2^, 50 s T(ii) λ780, CW, Spot 1 cm, near-contact, 60 mW, fluence 107.1 J/cm^2^, Irrad. 0.06 W/cm^2^, total energy 28 J, Beam dia 1.6 mm, 0.028 cm^2^, 33 s	2/2	CT CT + RT 660 > 780
Abramoff, 2008 [31]	RCT B Pre-treatment	14 Patients. T—7, C—7. Tx delivered 0.5 x day x 44 days. 7 sites @ 126 points.	λ685, CW, in contact, 35 mW, fluence 72 J/cm^2^, Irrad 11.67 W/cm^2^, total energy χ J, Beam dia 0.6 mm, 0.003 cm^2^, 54 s	1/3	CT
Kuhn, 2009 [32]	RCT DB Post-treatment	21 Patients. T—9, C—12. Tx delivered 1 x day x 5 days. χ sites @ χ points.	λ830, 100 mW, fluence 4 J/cm^2^, Irrad. χ W/cm^2^, total energy χ J, Beam dia χ, χ cm^2^, χ s	χ/1	CT
Khouri, 2009 [33]	RCT Pre-treatment	22 Patients. T—12, C—10 Tx delivered χ x day x χ days. χ sites @ χ points.	λ660 + 780, CW, in contact, 25 mW, fluence 6.9 J/cm^2^, Irrad. 0.69 W/cm^2^, total energy 5.4 J, Beam dia 2 mm, 0.036 cm^2^, 10 s	χ/3	CT CT + RT
Silva, 2011 [34]	RCT B Pre-treatment	42 Patients. T—21, C—21. Tx delivered 1 x day x 9 days. 10 sites @ 80 points.	λ660, CW, in contact, 40 mW, fluence 11.1 J/cm^2^, Irrad 1 W/cm^2^, total energy 12.8 J, Beam dia 2 mm, 0.036 cm^2^, 10 s	χ/3	CT CT + RT
Lima, 2012 [35]	RCT DB Pre-treatment	75 Patients. T—37, C—38. Tx delivered 1 x day x χ days. 9 sites @ χ points.	λ660, CW, in contact, 10 mW, fluence 2.8 J/cm^2^, Irrad. 0.25 W/cm^2^, total energy χ J, Beam dia χ, 0.036 cm^2^, 10 s	0/2	RT
Oton-Leite, 2012 [36]	RCT Pre-treatment	60 Patients. T—30, C—30. Tx delivered 1 x day x χ days. 11 sites @ 55 points.	λ685, CW, in contact, 35 mW, fluence 2 J/cm^2^, Irrad χ W/cm^2^, total energy χ J, Beam dia χ mm, χ cm^2^, χ s	χ/3	RT
Hodgson, 2012 [37]	RCT DB Post-treatment	80 Patients. T—40, C—40. Tx delivered 1 x day x 14 days. 3 sites @ χ points.	λ670, CW, in contact, χ mW, fluence 4 J/cm^2^, Irrad. 0.05 W/cm^2^, total energy χ J, Beam dia χ, χ cm^2^, 80 s	1/0	CT LED extraoral
Carvalho, 2011 [38]	RCT Pre-treatment	70 Patients. T(i)—35, T(ii) 35 C—0. Tx delivered 1 x day x χ days. 7 sites @ χ points.	T(i) λ660, CW, in contact, 5 mW, fluence 1.4 J/cm^2^, Irrad. 0.125 W/cm^2^, total energy χ J, Beam dia 2 mm, 0.036 cm^2^, 10 s T(ii) λ660, CW, in contact, 15 mW, fluence 4.2 J/cm^2^, Irrad. 0.375 W/cm^2^, total energy χ J, Beam dia 2 mm, 0.036 cm^2^, 10 s	1/1	RT RT + CT
Arbabi-Kalati, 2012 [39]	RCT DB Pre-treatment	48 Patients. T—24, C—24. Tx delivered χ x day x χ days. χ sites @10 points.	λ630, χ mode, χ contact, 30 mW, fluence 5 J/cm^2^, Irrad χ W/cm^2^, total energy χ J, Beam dia χ mm, χ cm^2^, χ s	3/2	CT
Antunes, 2013 [40]	RCT DB Pre-treatment	94 Patients. T—47, C—47. Tx delivered 1 x day x 5 days. 8 sites @ 72 points.	λ660, CW, in contact, 100 mW, fluence 4.2 J/cm^2^, Irrad 0.417 W/cm^2^, total energy 72 J, Beam dia 5 mm, 0.24 cm^2^, 10 s	2/3	CT + RT
Oton-Leite, 2013 [41]	RCT DB Pre-treatment	60 Patients. T—30, C—30. Tx delivered 1 x day x 30 days. 11 sites @ 54 points.	λ685, CW, 2 mm distance, 35 mW, fluence 23.9 J/cm^2^, Irrad 1.25 W/cm^2^, total energy 46.4 J, Beam dia 2 mm, 0.036 cm^2^, 25 s	2/2	RT
Oton-Leite, 2015 [42]	RCT DB Pre-treatment	30 Patients. T—15, C—15. Tx delivered 3 x day x 21 days. 5 sites @ 43 points.	λ660, CW, in contact, 25 mW, fluence 6.9 J/cm^2^, Irrad 0.625 W/cm^2^, total energy 10.75 J, Beam dia 2 mm, 0.036 cm^2^, 10 s	χ/3	RT + CT
Ferreira, 2016 [43]	RCT DB Pre-treatment	35 Patients. T—17, C—18. Tx delivered 1 x day x 5 days. 9 sites @ 27 points.	λ650, CW, in contact, 100 mW, fluence 3.57 J/cm^2^, Irrad 1.46 W/cm^2^, total energy 54 J, Beam dia 2 mm, 0.028 cm^2^, 20 s	1/2	CT
Amadori, 2016 [44]	RCT DB Post-treatment	123 Patients. T—62, C—61. Tx delivered 1 x day x 4 days. χ sites @ χ points.	λ830, 150 mW, χ mode, χ contact, 100 mW, fluence 4.5 J/cm^2^, Irrad 0.15 W/cm^2^, total energy χ J, Beam dia χ mm, 1 cm^2^, 30 s	1/0	CT
Elad, 2011 [45]	RCT DB Pre-treatment	19 Patients. T—10, C—9. Tx delivered 1 x day x 25 days. χ sites @ χ points.	λ650, χ mode, χ contact, χ mW, fluence χ J/cm^2^, Irrad 0.2 W/cm^2^, total energy χ J, Beam dia χ mm, χ cm^2^, 68 s	1/2	CT CT + RT LED
Gautam, 2013 [46]	RCT DB Pre-treatment	220 Patients. T—110, C—110. Tx delivered 1 x day x 32 days. 6 sites @ χ points.	λ632.8, CW, Spot 1 cm, 24 mW, fluence 3.5 J/cm^2^, Irrad 0.024 W/cm^2^, total energy 38 J, Beam dia 0.6 mm, 1 cm^2^, 125 s	3/3	CT+RT
Gautam, 2015 [3]	RCT DB Pre-treatment	46 Patients. T—22, C—24. Tx delivered 1 x day x 5 days. 12 sites @ χ points.	λ632.8, CW, Spot 1 cm, 24 mW, fluence 3 J/cm^2^, Irrad 0.024 W/cm^2^, total energy 36 J, Beam dia 0.6 mm, 1 cm^2^, 125 s	2/3	RT
Kelner, 2007 [47]	RCT B Pre-treatment	49 Patients. T—24, C—25. Tx delivered χ x day x χ days. χ sites @ χ points.	λ685, CW, in contact, 35 mW, fluence 1.1 J/cm^2^, Irrad χ W/cm^2^, total energy χ J, Beam dia χ mm, χ cm^2^, 32 s	χ/1	RT RT + CT
Marin-Conde, 2019 [48]	RCT DB Pre-treatment	41 Patients. T—26, C—15. Tx delivered χ x day x χ days. χ sites @ 72 points.	λ940, CW, in contact, 500 mW, fluence 83.3 J/cm^2^, Irrad 13.88 W/cm^2^, total energy 216 J, Beam dia 2 mm, 0.036 cm^2^, 6 s	χ/3	RT + CT
Vitale, 2017 [49]	RCT B Post-treatment	16 Patients. T—8, C—8 Tx delivered 1 x day x χ days. χ sites @ χ points.	λ970, CW, scanning defocussed, 1600 mW, fluence χ J/cm^2^, Irrad 1.6 W/cm^2^, total energy χ J, Beam dia 2 mm, 1 cm^2^, 240 s	1/2	CT Full mouth
Salvador, 2017 [50]	RCT B Pre-treatment	51 Patients. T—27, C—24. Tx delivered 1 x day x χ days. χ sites @ 80 points.	λ660, CW, in contact, 40 mW, fluence 4 J/cm^2^, Irrad 1 W/cm^2^, total energy 12.8 J, Beam dia 2 mm, 0.04 cm^2^, 4 s	?/3	CT
Gobbo, 2018 [51]	RCT BPost-treatment	101 Patients. T—51, C—50. Tx delivered 1 x day x 4 days. 9 sites @ χ points.	λ660 + 970, gated 50% duty cycle, scanning defocussed, 320 mW, fluence 36.8 J/cm^2^, Irrad 0.32 W/cm^2^, total energy 144 J, Beam dia χ mm, 1 cm^2^, 450 s	1/3	CT/RT Full mouth
